# *clbP* Gene, a Potential New Member of the β-Lactamase Family

**DOI:** 10.3390/ijms232415642

**Published:** 2022-12-09

**Authors:** Adel Azour, Charbel Al-Bayssari, Lucile Pinault, Saïd Azza, Jean-Marc Rolain, Seydina M. Diene

**Affiliations:** 1 Faculty of Sciences 3, Michel Slayman Tripoli Campus, Lebanese University, Ras Maska 1352, Lebanon; 2 MEPHI, IRD, APHM, IHU-Méditerranée Infection, Faculté de Pharmacie, Aix Marseille University, 13005 Marseille, France; 3 Department of Medical Laboratory Sciences, Faculty of Health Sciences, University of Balamand, Tripoli P.O. Box 100, Lebanon

**Keywords:** antibiotics, *clbP* gene, bacterial toxins

## Abstract

The colibactin island (*pks*) of *Escherichia coli* formed by 19 genes (55-Kb), encodes non-ribosomal peptide (NRP) and polyketide (PK) synthases, which allow the synthesis of colibactin, a suspected hybrid PK-NRP compound that causes damage to DNA in eukaryotic cells. The *clbP*, an unusual essential gene, is found in the operon structure with the *clbS* gene in the *pks*-encoded machinery. Interestingly, the *clbP* gene has been annotated as a β-lactamase but no previous study has reported its β-lactamase characteristics. In this study, we (i) investigated the β-lactamase properties of the *clbP* gene in silico by analysing its phylogenetic relationship with bacterial β-lactamase and peptidase enzymes, (ii) compared its three-dimensional (3D) protein structure with those of bacterial β-lactamase proteins using the Phyr2 database and PyMOL software, and (iii) evaluated in vitro its putative enzymatic activities, including β-lactamase, nuclease, and ribonuclease using protein expression and purification from an *E. coli* BL21 strain. In this study, we reveal a structural configuration of toxin/antitoxin systems in this island. Thus, similar to the toxin/antitoxin systems, the role of the *clbP* gene within the *pks*-island gene group appears as an antitoxin, insofar as it is responsible for the activation of the toxin, which is colibactin. In silico, our analyses revealed that ClbP belonged to the superfamily of β-lactamase, class C. Furthermore, in vitro we were unable to demonstrate its β-lactamase activity, likely due to the fact that the *clbP* gene requires co-expression with other genes, such as the genes present in the *pks*-island (19 genes). More research is needed to better understand its actions, particularly with regards to antibiotics, and to discover whether it has any additional functions due to the importance of this gene and its toxicity.

## 1. Introduction

Natural products, such as polyketide (PK), non-ribosomal peptide (NRP) and hybrid NRP-PK, are all promising candidates for therapeutic development [1]. These natural products, which have a wide range of biological activities, can include antibiotics, pigments, immunosuppressants, cytostatic toxins and siderophores [2,3]. Some micro-organisms (e.g., bacteria) produce a natural toxin or antibiotic to control their ecosystems [4]. Furthermore, antibiotics are grouped based on their mode of action, including proteins, cellulose synthesis inhibitors, DNA, RNA and many others [5,6]. These micro-organisms use mechanisms of auto-resistance to protect their bioactive biosynthetic compounds from attacks. These mechanisms are similar to toxin–antitoxin systems [5,7]. Among these antibiotics, thienamycin is encoded by a thienamycin gene cluster (TGC). This cluster is made up of 22 genes (*thnA* to *thnY*) from the *Streptomyces cattleya* NRRL 8057 genome. In addition to thienamycin and within the same *S. cattleya* NRRL 8057 genome, a cephalosporin gene cluster (CGC) encodes cephamycin (cephalosporin subclass). This cluster is made up of 16 genes [8]. Recently, Diene et al., revealed a toxin/antitoxin system in thienamycin gene cluster and reported that the *thnS* gene, which is part of this cluster, appears as a class B metallo-β-lactamase enzyme [9]. In addition to its β-lactam hydrolysis activity, the *thnS* protein appears to have an RNase/DNase activity and represents an essential antidote for the bacterium *S. cattleya* against its biosynthesised poison, thienamycin [9]. β-lactam are a group of bactericidal antibiotics, which inhibit the synthesis of the peptidoglycan layer of bacterial cell walls. The peptidoglycan layer is a crucial component for the bacteria, giving structural strength to the cell wall, as well as protecting bacteria against osmotic pressure. The final transpeptidation step in the synthesis of the peptidoglycan is controlled by DD-transpeptidases, also known as penicillin binding proteins (PBPs). β-lactams inhibit peptidoglycan synthesis by binding to these PBPs [10,11].

In 2006, Nougayrède et al., discovered a huge (54 kb) genomic island termed *pks* in *Escherichia coli* strains and other *Enterobacteriaceae*, which encodes a characteristic cluster of NRP synthases (NRPS), PK synthases (PKS) and hybrid NRPS/PKS [12]. The *pks*-island has been found in *E. coli* strains that cause extraintestinal illnesses, such as urinary tract infections and septicaemia, as well as commensal strains from healthy subjects [12,13,14,15,16,17,18,19,20]. The *pks*-island encodes for the manufacture of a PK-NRP hybrid molecule known as colibactin, according to genomic and functional investigations [21]. During infection, *E. coli* strains that contain this gene cluster cause DNA double-strand breaks in human eukaryotic cells [12,22]. The *pks*-island is made up of 19 genes ranging from *clbA* to *clbS* (*clbS* is the gene responsible for colibactin resistance), which are involved in colibactin production [12,15,23,24]. The *pks*-island encodes nine auxiliaries, tailoring, and editing enzymes, in addition to eight NRP and PK mega synthases [12,18,25]. Eight of these proteins are necessary to cause DNA damage in infected cells, including ClbP [25]. Furthermore, despite annotation of the *clbP* gene as β-lactamase (Ac. number: CDO15367), this activity remains unreported.

Here, we aim (i) to investigate the phylogenetic relationship of the ClbP enzyme with known bacterial β-lactamases, (ii) to compare its three-dimensional (3D) protein structure with bacterial β-lactamases, and then (iii) to evaluate whether or not the enzymatic activity on various substrates, including antibiotics, DNA and rRNA molecules, exists.

## 2. Results

### 2.1. In Silico Visualisation of the clbP Gene and Enzyme

#### 2.1.1. *clbP* Gene and Origin of the Enzyme

**Figure 1:**
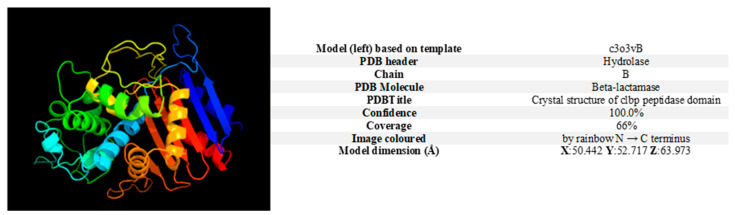
A 3D model of the ClbP enzyme was created using the Phyre2 web portal for protein modelling, prediction, and analysis (http://www.sbg.bio.ic.ac.uk/phyre2/html/page.cgi?id = index (accessed on 22 November 2021)).

The colibactin gene cluster (*pks*-island) spans 55 kb and contains 19 genes ranging from *clbA* to *clbS* one of which is *clbP* [12]. According to Phyre2, the presentation of the proteolytic sequence of the *clbP* gene was based on template c3o3yB, with PDB header: hydrolase, chain: B, and the best homologous sequence was a β-lactamase (confidence: 100%, coverage: 66%) (Figure 1).

#### 2.1.2. The ClbP Enzyme’s Phylogenetic Link to Bacterial β-Lactamases

**Figure 2:**
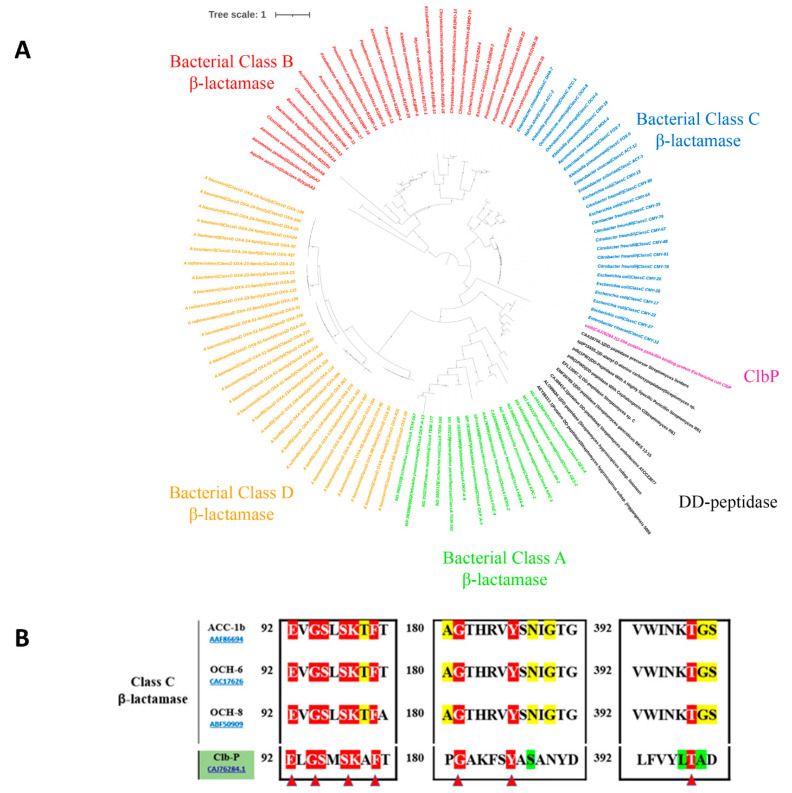
(**A**) Phylogenetic tree of pre-colibactin peptidase ClbP with described bacterial β-lactamase sequences. (**B**) Multiple sequence alignment was performed among class C β-lactamases and the ClbP sequence. Three characteristic motifs (motif I, II, and III) with significant catalytic residues are shown. Highly conserved catalytic residues are shown in red triangles.

As shown in Figure 2A, the ClbP protein appears closely related to the bacterial class C β-lactamases, according to the inferred phylogenetic tree with the four bacterial classes of β-lactamases (A, B, C, and D) and the DD-peptidase sequences. Alignment of the ClbP sequence with the other three class C β-lactamase proteins, ACC-1b (AAF86694), OCH-6 (CAC17626), and OCH-8 (ABF50909) confirms its membership, emphasising the conserved “SxxK” motif, which is the essential catalytic residue for the enzymatic activity of this class. In the same way, the second conserved motif 186YAS versus YxN was also found (Figure 2B).

**Figure 3:**
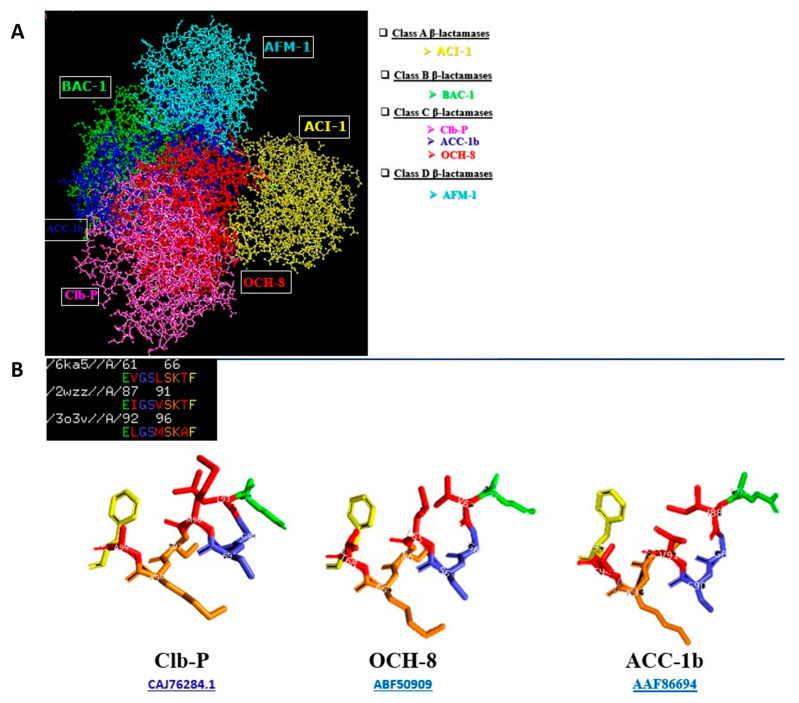
(**A**) A molecular model of the ClbP protein exhibiting a protein structure similarity with class C β-lactamase proteins. (**B**) 3D structure of the complete motif “SxxK” created using the PyMOL 1.8.6.0 software shown in the ClbP enzyme and in the other class C β-lactamases.

Furthermore, comparison of 3D structures of the ClbP with other protein sequences of β-lactamases of different A, B, C, and D classes showed the association of the ClbP with the class C β-lactamase genes (Figure 3A). The 3D structure of this complete “SxxK” motif created using the PyMOL 1.8.6.0 software showed a similar 3D structure between the ClbP enzyme and class C β-lactamase genes (Figure 3B).

**Figure 4:**
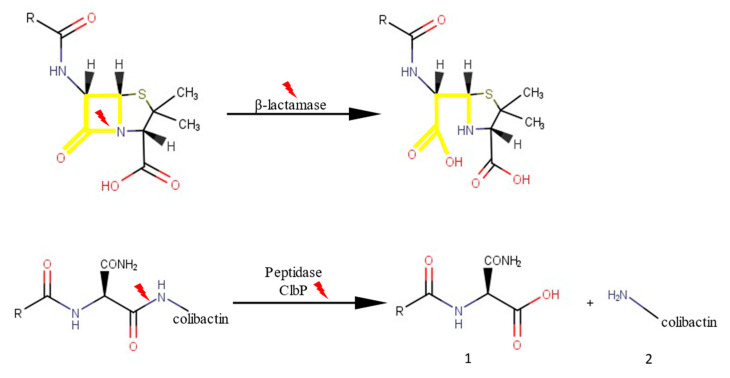
Comparison between chemical cleavage of β-lactamase and the *clbP* gene. The chemical structure was creating using MarvinView version 22.1.0.

Moreover, the chemical cleavage site of the β-lactamase and the ClbP enzyme are similar; both enzymes cut the bond between nitrogen and carbon atoms (Figure 4).

**Figure 5:**
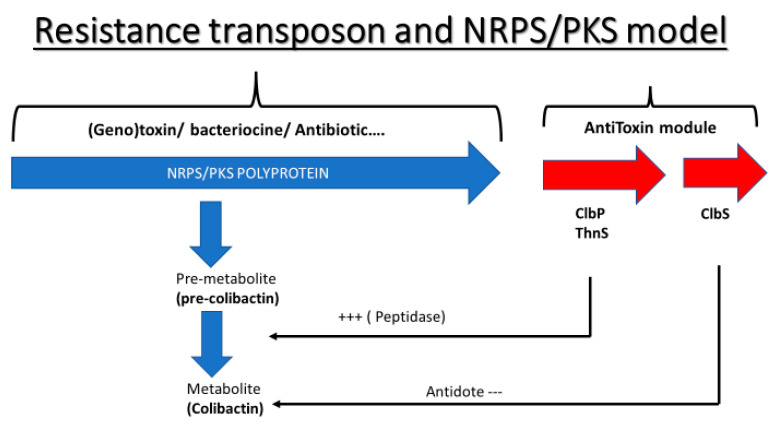
The schematic representation of the NRPS/PKS colibactin cluster as a toxin/antitoxin system. The first part of the cluster (in blue) composed by the sixteen first genes encode for the pre-colibactin metabolite (toxin) while the second part of this cluster (in red) represents the antitoxin module that acts as protector against the synthetized metabolite. (+++: Transformation of pre-colibactin to activated colibactin; ---: Inactivation of colibactin).

The colibactin cluster was considered to be part of a system similar to the toxin/antitoxin system (Figure 5). This group of genes is genetically made up of an NRPS/PKS polyprotein set which synthesizes pre-colibactin (inactive toxin) as a pre-metabolite. The activity of the *clbP* gene, encoding a peptidase, is similar to that of beta-lactamase, which is encoded by the *thnS* gene. In addition, the *clbP* gene converts pre-colibactin into active colibactin (active toxin) (metabolite). Finally, the *clbS* gene, which encodes an antidote protein, destroys colibactin so that it will be inactive and so the *clbS* gene protects the cell from the effect of this toxin.

### 2.2. In Vitro Expression of the clbP Gene

#### 2.2.1. β-Lactam-Hydrolysing Activity

The enzymatic activity of the ClbP produced was evaluated against various antibiotic substrates, including nitrocefin, penicillin G, amoxicillin, and ampicillin, to determine their enzymatic activity. Results showed that ClbP was unable to increase *E. coli* resistance to different β-lactam antibiotics and was unable to hydrolyse the nitrocefin substrate. The minimum inhibitory concentrations (MICs) of *E. coli* harbouring the *clbP* gene against penicillin G, amoxicillin, and ampicillin were 16 µg/mL, 2 µg/mL, and 0.75 µg/mL, respectively.

#### 2.2.2. Nuclease and Ribonuclease Activity

**Figure 6:**
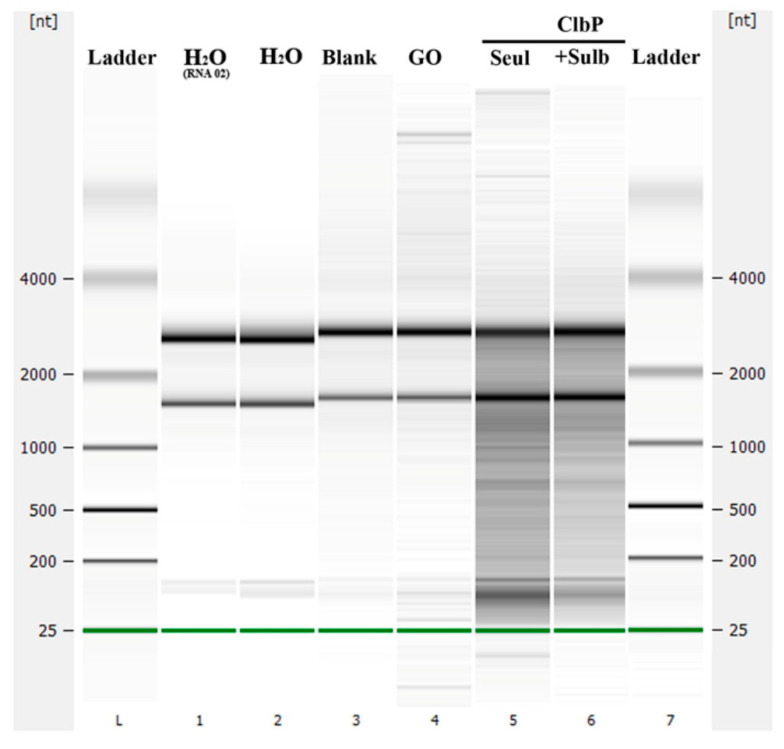
Test of the RNAse activity of the purified ClbP enzyme. GO (glycine oxidase) is used here as negative control enzyme.

The ClbP nuclease and ribonuclease activities were studied. After two hours of incubating the ClbP enzyme with extracted *E. coli* RNA in the presence or absence of a β-lactamase inhibitor (i.e., sulbactam), results showed that *E. coli* RNA was not hydrolysed. The negative control enzyme (glycine oxidase) did not hydrolyse the *E. coli* RNA, while with the positive control (RNAse), the *E. coli* RNA was hydrolysed (Figure 6).

**Figure 7:**
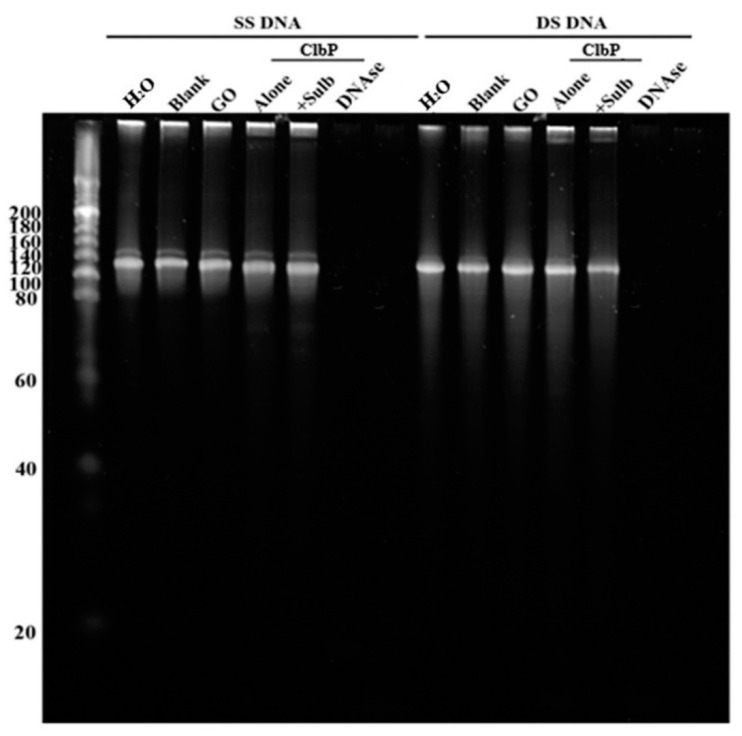
Test of the DNAse activity of the purified ClbP enzyme. GO (glycine oxidase) is used here as negative control enzyme.

The ClbP was also incubated with synthetic single-stranded and double-stranded DNA to test its DNAse activity. Results showed that the ClbP did not digest the double-stranded or the single-stranded DNA (Figure 7); the same results were observed with the negative control enzyme (GO); however, the single-stranded and double-stranded DNA were hydrolysed by the positive control enzyme (DNAse).

## 3. Discussion

In the primordial war of microbes to colonise and govern their habitats, natural β-lactam antibiotics, such as cephalosporin C, thienamycin and penicillin [26,27] are expressed as poisons. β-lactamase enzymes seem to be antidotes to the β-lactams produced by bacteria as a means of self-defence against these bioactive chemicals in this setting. This shows that as with the toxin/antitoxin systems, genes encoding for β-lactams and β-lactamases have co-evolved in the NRPS/PKS clusters of micro-organisms. It might, therefore be assumed that the widespread use and overuse of antibiotics, such as β-lactams by humans over the last century has altered this co-evolution, forcing bacteria to develop β-lactamases. The *clbP* gene, which is part of the colibactin gene cluster (55-Kb), is responsible for the production and expression of the colibactin drug by the *E. coli* IHE3034 isolate and is an important component of colibactin’s prodrug resistance mechanism [28,29,30,31]. Furthermore, studies have shown that ClbP is a member of a novel subfamily of extra cytoplasmic serine-reactive peptidases that operate as NRP compound maturation enzymes. The critical role of ClbP in the formation of the biological impact makes it a key target for controlling the bioactivity of the PK-NRP-producing *pks* gene cluster, which may affect commensalism and/or pathogenicity and serve as a risk factor for the development of colorectal cancer [15,18,28,31,32]. In this study, the *clbP* gene was evaluated in silico and expressed in vitro in order to categorise its β-lactamase activity. We managed to show that the *clbP* gene has the same chemical cleavage as β-lactamase at the level of the azote-carbon bond, and were also able to show in silico that the *clbP* gene has all the characteristics of a β-lactamase. The sequence of the ClbP protein appears to be closely related to class C β-lactamase on the phylogenetic tree. Moreover, the sequence of the ClbP protein showed the present of the unique motif characterising the β-lactamase class C. Finally, the protein sequence of ClbP and the four β-lactamase classes (A, B, C, and D) analysed by PyMOL showed a clear match between ClbP and the class C β-lactamase. All of these in silico results clearly show that ClbP has a peptidase which is closely related to class C β-lactamase. In contrast, the expression of ClbP in vitro did not show a clear β-lactamase activity for this gene. Furthermore, investigations of β-lactamase, specifically class D, showed RNAse/DNAse functions [9]. In addition, all of the studies have demonstrated that in the absence of the *clbP* gene which codes for the ClbP enzyme in the *pks*-island, DNA degradation is reduced, however in the presence of *clbP* in this island, DNA degradation is increased [32,33]. Our investigations show that ClbP did not show any DNAse/RNAse activity. This is the first study that presents the characteristics, the phylogenetic relationship and the comparison of the three-dimensional (3D) protein structure of the ClbP enzyme with bacterial β-lactamases similarities, and its evaluation of the enzymatic activity on various substrates, including antibiotics.

## 4. Materials and Methods

### 4.1. Phylogeny of the ClbP Protein

The ARG-ANNOT database was used to extract representative protein sequences from the four described β-lactamase classes (A, B, C, and D) [34] and grouped together to form a phylogenetic tree to investigate the phylogenetic relationship of the ClbP from the *E. coli* IHE3034 genome with known bacterial β-lactamases. In addition, certain DD-peptidase sequences that are more closely related to β-lactamase class C were downloaded from the NCBI database and incorporated into the phylogenetic tree analysis. Mafft [35] was used to align all the protein sequences, Trimal [36] was used to repair gaps in the alignment, and FastTree [37] was used to construct the phylogenetic tree.

### 4.2. Structural Prediction and Alignment of ClbP

The ClbP sequence (CAJ76284.1) was uploaded to the Phyre2 web portal for protein modelling, prediction, and analysis (http://www.sbg.bio.ic.ac.uk/phyre2/html/page.cgi?id=index (accessed on 12 January 2022)).

Using the PyMOL 1.8.6.0 software, the molecular structure (ball and sticks) of β-lactamase gene sequences, as well as the 3D shape of β-lactamase motifs in the ClbP sequence and other β-lactamase sequences, were visualised. Furthermore, multiple sequence alignment with hierarchical clustering (http://multalin.toulouse.inra.fr/ (accessed on 2 February 2022)) was used for multiple sequence alignment of distinct β-lactamase sequences.

### 4.3. Recombinant Protein Expression and Purification

The ClbP (CAJ76284.1) protein was designed with a Strep-tag at the N-terminus. Synthetic genes optimised for expression in *E. coli* were purchased from GenScript (Piscataway, NJ, USA) and each construct was cloned into a pET24a (+) plasmid. Recombinant proteins were expressed in *E. coli* BL21(DE3)-pGro7/GroEL (Takara, Kyoto, Japan) cultured in ZYP-5052 Medium. L-arabinose (0.2 percent *m*/*v*) was added to induce the expression of chaperones and the temperature was then reduced to 20 °C when the culture reached an OD_600 nm_ = 0.6 at 37 °C. Twenty hours later, centrifugation was used to collect the cells (5000× *g*, 30 min, 4 °C), and the pellet was resuspended in 50 mM Tris pH 8, 300 mM NaCl, 0.25 mg/mL lysozyme, 10 g/mL DNAse I, 0.1 mM PMSF and kept at −80 °C overnight [38], as previously described. After thawing, three successive cycles of sonication (30 s, amplitude 45) using a Q700 sonicator system (QSonica) were used to disrupt partially lysed cells. Following centrifugation (11,000× *g*, 20 min, 8 °C), cellular debris was removed [38]. Recombinant proteins were purified using a 5 mL StrepTrap HP column on an AKTA avant system (GE Healthcare, Chicago, IL, USA) with elution buffer: 50 mM Tris pH 8, 300 mM NaCl, 2.5 mM desthiobiotin. The purity of the collected proteins was determined using SDS-PAGE analysis with Coomassie staining [38]. MALDI-TOF MS analysis of gel bands previously obtained by SDS-PAGE was used to confirm protein expression. A Nanodrop 2000c spectrophotometer (ThermoScientific, Madison, WI, USA) was used to quantify protein concentrations [38].

### 4.4. Antibiotic Sensitivity Testing

For β-lactam antibiotic susceptibility testing, each *E. coli* with OD = 0.6, 0.01 g/mL of IPTG (isopropylthiogalactoside) (Euromedex, Strasbourg, France) was added to 1 mL of the broth to induce protein expression. Then, 0.5% McFarland bacterial solution was prepared and grown on MH agar (Muller Hinton) (BioMérieux, Marcy l’Étoile, France). The MIC of penicillin G, amoxicillin, and ampicillin has been determined using the E test method (BioMérieux, Marcy l’Étoile, France), according to the European Committee on Antimicrobial Susceptibility Testing (https://www.eucast.org (accessed on 6 March 2022)). After 24 h of incubation, the results were visualized. As negative controls, uninoculated and non-transformed *E. coli* BL21 cultures and control cultures without β-lactam antibiotics were used.

### 4.5. Ribonuclease and Nuclease Activity

Each polynucleotide or polyribonucleotide (1 µg) was incubated with 10 µg of pure ClbP alone or in the presence of penicillinase inhibiter (10 mM of Sulbactam) in a final volume of 15 µL for two hours at 37 °C. Single-stranded DNAs were synthesized polynucleotides [8], and double-stranded DNA (130-bp) was made by annealing positive and negative single-stranded DNAs (130-bp) in a thermocycler for two hours at temperatures reducing from 95 to 25 degrees Celsius. CutSmart Buffer (New England Biolabs, 50 mM Potassium Acetate, 20 mM Tris–acetate, 10 mM Magnesium Acetate, and 100 g/mL BSA pH 7.9) was used for enzymatic reactions, which were carried out at 37 °C for two hours. Following incubation, the sample was placed onto a 12% denaturing PolyAcrylamide Gel Electrophoresis (dPAGE). *Bacillus subtilis* Glycine Oxidase (GO), a negative enzyme control, was produced and purified under the same conditions as ClbP and evaluated. A positive control enzyme, Turbo DNAse, was used at a concentration of 1 U. The RNA from *E. coli* was isolated using RNeasy columns to test ribonuclease activity (Invitrogen, Carlsbad, CA, USA). The integrity of the RNA preparations was analysed on an Agilent Bioanalyzer 2100, using an Agilent RNA 6000 Pico LabChip. They were found to be of high integrity, with an RNA Integrity Number (RIN) = 10. The enzymatic reactions were carried out by incubating 1 µg of RNA in CutSmart Buffer with 10 µg of pure ClbP alone and in the presence of a penicillinase inhibitor for two hours at 37 °C in a final volume of 15 µL. The RNA 6000 Pico LabChip was used to monitor (visualise) RNA-hydrolysing activity after incubation (Agilent 2100 Bioanalyzer). The glycine oxidase of *B. subtilis* was also used as a negative control enzyme.

## 5. Conclusions

Our study showed that the toxin/antitoxin system exists in the *pks*-island. In addition, our results showed that the *clbP* gene, with its peptidase activity in this cluster, appears to be closely related to bacterial class C β-lactamases enzymes. However, we were unable to establish its β-lactamase activity in vitro, possibly because the *clbP* gene requires co-expression with other genes, such as the genes present in the *pks*-island (19 genes), a condition which we did not meet in our study because we only expressed the *clbP* gene. Due to the importance of this gene on various levels, further research is needed to better understand its functions, particularly with regard to antibiotics, and to see whether it has any more functions.

## Data Availability

Not applicable.
